# Supplementary material for: A Role for ER-Beta in the Effects of Low-Density Lipoprotein Cholesterol and 27-Hydroxycholesterol on Breast Cancer Progression: Involvement of the IGF Signalling Pathway?
Source: Cells. 2021 Dec 29;11(1):94. doi: 10.3390/cells11010094 (PMC8749996; doi:10.3390/cells11010094)
Supplement: Supplementary file 1 [file cells-11-00094-s001.zip › cells-1377602-supplementary.pdf]

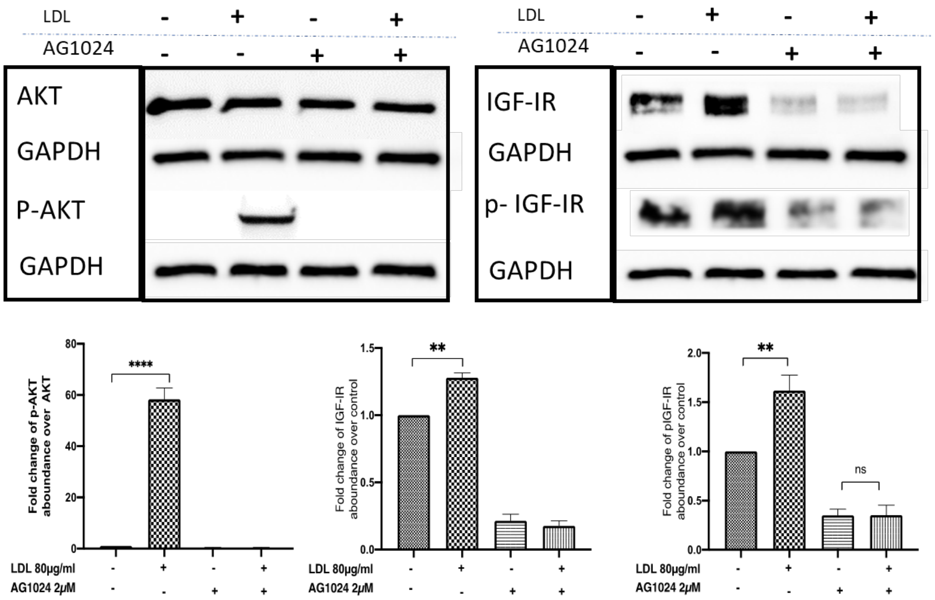

Supplementary Figure S1: Levels of the IGF-IR, p-IGF-IR, AKT, p-AKT were determined using western blotting in the presence or absence of LDL with or without an insulin-like growth factor receptor antagonist: Representative western blots show MCF-7 cells, treated with LDL (80µg/ml) in the presence or absence of a tyrosine kinase inhibitor (AG1024, 2µM) for 48 hours. Densitometry analysis of fold changes of IGF-IR against loading control GAPDH, and p-AKT and p-IGF-IR relative to AKT and the IGF-IR respectively. Data representative of mean ±SEM (n=3). P-values were determined by using GraphPad Prism one-way Statistical analysis: ANOVA test plus least significant difference (LSD) post-hoc test (*p < 0.05, **p < 0.01, ***p < 0.001).
